# Prevalence of left-handers and their role in antagonistic sports: beyond mere counts towards a more in-depth distributional analysis of ranking data

**DOI:** 10.1098/rsos.250303

**Published:** 2025-09-24

**Authors:** Tim Simon, Florian Loffing, Elisa Frasnelli

**Affiliations:** ^1^ CIMeC Center for Mind/Brain Sciences, University of Trento, Rovereto (TN), Italy; ^2^ German Sport University Cologne, Cologne, NRW, Germany

**Keywords:** left-handedness, sports, distributional analysis, negative frequency-dependent advantage hypothesis, innate superiority hypothesis

## Abstract

Approximately 10% of the general population is left-handed, yet a disproportionately higher percentage of left-handers is observed among athletes in various sports, including combat sports and interactive ball games. This overrepresentation is generally considered evidence of a performance advantage. However, previous studies have primarily focused on simple calculations of left-hander proportions within larger sport populations, without examining their distribution across different performance levels. Our study advances the research by conducting more in-depth distributional analyses of left-hander frequencies across various performance tiers in various sports, including fencing (épée, foil, sabre) and interactive ball games (table tennis, tennis, badminton). Our findings for fencing and table tennis reveal an average overrepresentation of left-handers across performance levels, with notably higher proportions at upper echelons. This strengthens the idea of a performance advantage for left-handedness in certain antagonistic sports beyond the evidence inferred from the traditional performance-independent analysis of overrepresentation. Left-handers’ relative athletic success is typically attributed to their opponents' unfamiliarity with left-handed action patterns due to the relative rarity of left-handers in the general population (negative frequency-dependent advantage hypothesis). However, we also raise the question of whether left-handers’ edge may partially stem from other, frequency-independent factors (innate superiority hypothesis).

## 1. Introduction

Handedness, i.e. the preference to use one hand over the other for performing certain motor tasks, is the most widely investigated form of behavioural asymmetry in humans [1]. In research, individuals’ handedness is commonly assessed using standardized self-report questionnaires, such as the Edinburgh Handedness Inventory (EHI), which asks individuals which hand they prefer for a range of everyday activities (e.g. writing, drawing, using scissors, brushing teeth, throwing an object, etc.) [2]. On all continents, the large majority of the human population is right-handed, while a minority is left-handed, with the global prevalence of left-handedness estimated at approximately 10% [3].

However, in various sports, athletes who use their left hand for sport-specific unimanual actions—such as wielding a one-handed weapon or striking a ball with a racket—are overrepresented relative to the approximately 10% prevalence of left-handedness in the general population. In such sports, which require unilateral use of either the left or right hand for task-specific actions, athletes’ sport-specific hand preferences typically align with their everyday handedness, as assessed by tools like the EHI; by contrast, in sports involving bimanual tasks that require coordinated use of both hands—such as golf, baseball (batting) or field and ice hockey—sport-specific hand use is often not clearly correlated with everyday handedness [4]. Notably, in unimanual sports, an overrepresentation of left-handers has been reported, particularly in disciplines involving antagonistic, duel-like interactions, such as combat sports. In épée and foil fencing, the percentage of athletes in the top-200 year-end world rankings from 2007 to 2016 who used their left hand to wield the weapon ranges from 16.85 to 22.77% among female fencers and from 20.86 to 25.75% among male athletes [5]. A previous study reported even higher percentages among French elite fencers, with left-handers comprising 47.2–50% of those ranked in the top eight and top four, respectively, in men’s foil between 1979 and 1998 [6]. In 2019, 12.50% of professional female boxers (*n* = 1314) and 17.00% of male boxers (*n* = 10445) used the ‘southpaw stance’, characterized by positioning the right leg and shoulder closer to the opponent [7]. This stance is often recommended for athletes who are stronger with their left hand, as it facilitates more forceful left-handed punches. An overrepresentation of left-handers has also been reported in interactive ball games. In table tennis, between 18.64 (females) and 25.18% (males) of athletes ranked in the top-200 in the year-end world rankings between 2007 and 2016 wielded the racket in their left hand [5]. In cricket and baseball, 21.78−30.39% of male elite bowlers/pitchers, who were among the top rankings in major leagues between 2009 and 2014, used their left hand to throw the ball towards the opposing team’s batter/striker [8].

The overrepresentation of left-handed athletes is considered indirect evidence for a competitive edge [9–11]. Two main hypotheses have been proposed to explain this advantage. The *negative frequency-dependent advantage hypothesis (NFDA*) suggests that in antagonistic contests, left-handers benefit from their rarity in the general population: opponents are less familiar with left-handed action patterns, giving left-handers an element of surprise [10,12,13]. Negative frequency-dependent selection, a central concept in evolutionary biology, posits that the fitness of a trait decreases as it becomes more common and increases when it is rare. Applying this concept to sports, *NFDA* predicts that the less frequently athletes encounter left-handers, the greater the left-handers’ advantage—an advantage that diminishes as left-handers become more common and opponents gain experience.

By contrast, the *innate superiority hypothesis (IS*) proposes that left-handers enjoy a performance edge regardless of their frequency or opponents’ experience [11]. According to *IS*, various frequency-independent predispositions (potentially) associated with left-handedness may enhance athletic performance. For instance, left-handers might benefit from functional specializations of the right hemisphere of the brain [11]. Specifically, actions performed with the left hand are mainly controlled by the brain’s right hemisphere, which is dominant for visuospatial, spatiotemporal and visuomotor processing [14–18]. In (sport-relevant) tasks demanding these abilities, neural processing may be more efficient for left- compared to right-hand actions, potentially due to reduced information transfer delay [19–21]. By contrast, for right-hand actions, which are primarily controlled by motor networks in the contralateral left hemisphere, task-relevant commands require cross-hemispheric communication via the corpus callosum—possibly resulting in performance-hindering delays in time-critical scenarios [14,22,23]. Previous research also suggests that eye preference (i.e. the tendency to rely more on one eye than the other for visual input, especially when performing tasks that require visual alignment or precision, such as aiming, looking through a keyhole or microscope) modulates visuospatial attention [24]. In this context, it has been proposed that left-handers with right-eye dominance may benefit from the advantage of having the same right hemispheric control of their dominant left hand and visuospatial attention processing [21]. Right-hemisphere specialization has also been proposed to support more effective left-hand movements in precision-based tasks, such as aiming activities like fencing [14,25,26], as well as in object manipulation during close-distance contests, as seen in various combat sports [27,28]. Moreover, left- and right-handers use different strategies in recruiting brain networks for motor control during hand use, which may help explain left-handers’ advantages in certain sports [29]. According to the *hybrid control scheme*, hand use is associated with two distinct processes of motor control [30–32]. This model suggests that the hemisphere contralateral to the dominant hand (i.e. left in right-handers and right in left-handers) is specialized for predictive control of limb and task dynamics that allows the specification of motions that can be optimized for maximum speed, trajectory parameters, power and efficiency. This type of control, however, depends on the predictive ability and consistency of the mechanical environment. By contrast, the hemisphere ipsilateral to the dominant hand (i.e. right in right-handers and left in left-handers) appears specialized for impedance control processes that can stabilize performance and reduce errors in the face of unexpected mechanical conditions. While everyday tasks often split these roles between both hands—such as when cutting bread or hammering a nail—many sports demand both types of control from a single arm. For example, in fencing, the dominant arm is used both for thrusting-type motions that are expected to recruit the contralateral hemisphere (predictive control) and blocking motions that impede forces imposed by the opponent and are expected to recruit the ipsilateral hemisphere (impedance control). Interestingly, left-handers show greater recruitment of the ipsilateral cortex during dominant hand use than right-handers. This increased activation may reflect the need for left-handers to adapt to a right-handed world, often using their non-dominant right hand for dominant hand tasks, such as opening doors, using scissors or shaking hands. This idea is supported by previous research showing that experience and practice can enhance ipsilateral hemisphere recruitment during non-dominant hand use [33]. If left-handers learn to recruit the ipsilateral cortex to a greater degree than right-handers, this might contribute to left-handers’ relative success in certain sports: left-handers would likely have an inherent advantage in learning to recruit both hemispheres for complex sport-specific unimanual tasks [29]. Furthermore, *IS* posits that left-handers may benefit from certain hormonal profiles that are considered advantageous for sporting performance [34–37]. One candidate is testosterone, which has been associated with increased aggression and a greater tendency to engage in competitive behaviour [38]. Faurie *et al*. [39] reported higher testosterone levels in left-handers compared to right-handers. However, these concentrations were not directly linked to athletic performance, leaving it unclear whether differences in testosterone levels between left- and right-handers contribute to performance differences. Conceptually, *NFDA* and *IS* are not mutually exclusive; they may reflect complementary mechanisms contributing to left-handers’ competitive success.

**Figure 1.**
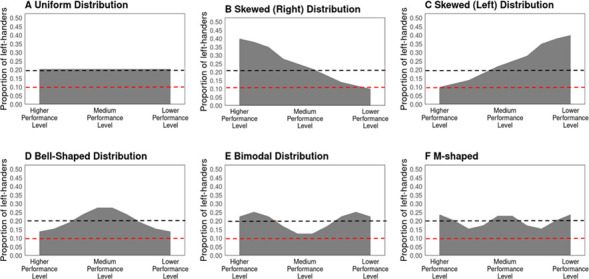
Examples of different possible distributions of left-handed athletes (A–F). Each scenario illustrates an overall overrepresentation of left-handed athletes, maintaining an average prevalence of 20%, as indicated by the black dashed line. This exceeds the general population average of approximately 10%, denoted by the red dashed line. The distributions of left-handers can vary significantly among different performance levels: higher, medium and lower.

Previous findings on the overrepresentation of left-handed athletes primarily rely on simple calculations of their proportion within larger sport populations. However, exclusively comparing frequencies of left-lateralized athletes to general population estimates may overlook potential nuances in the relationship between left-handedness and sport performance, leading to oversimplified conclusions. To illustrate, consider the following scenario. If 20% of athletes in a sport predominantly use their left hand for sport-specific activities—such as wielding a weapon or holding a racket—statistical tests would indicate an overrepresentation of left-handers, given their approximate 10% occurrence in the general population. In line with previous literature, this could lead one to infer that left-handed athletes possess a competitive advantage in this sport. However, without a deeper examination of how these athletes are distributed among various performance levels, such conclusions might be considered premature and unjustified. A proportion of 20% left-handers in a larger sport population is equally compatible with different distributions of left-handers across different skill levels. Left-handers could be uniformly distributed across proficiency levels (figure 1A); they might be concentrated more at the highest or lowest performance tiers (figure 1B,C) or around the middle tiers (figure 1D). Other patterns might include a bimodal distribution, where left-handers cluster at both the highest and lowest levels (figure 1E), or even more complex distributions (figure 1F).

A uniform distribution (figure 1A) in which left-handed athletes are consistently overrepresented across various performance levels, compared to general population estimates, would support the conclusion that left-handedness is linked to a general competitive performance edge in this sport. Similarly, a left-skewed distribution, with increasingly larger proportions of left-handers at higher skill levels than in the general population, aligns with the idea of a performance advantage for left-handedness (figure 1B). By contrast, other possible distributions (figure 1C–F) do not straightforwardly support a link between performance and left-handedness, indicating the need for revised theories concerning the role and performance of left-handers in sports. Therefore, distributional analyses are crucial to gain a deeper understanding of left-handedness in sports.

The call for a more in-depth analysis may also hold value and yield fruitful insights for sports like badminton or squash, where the overall prevalence of left-handers does not exceed general population estimates [5,8]. In such sports, left-handedness is usually not considered performance-relevant. However, without a closer examination of how left-handedness is distributed across performance categories, concluding that handedness has no relevance for athletic performance may be premature. If most left-handed athletes are concentrated in higher performance tiers, with a prevalence exceeding the general population level, handedness might indeed play a performance-relevant role in these sports.

So far, only a few studies have compared left-hander frequencies across performance levels. For instance, Azémar [6] observed a significant overrepresentation of left-handers at the highest performance levels in French elite fencing. Specifically, he reported that from 1979 to 1998, the proportion of left-handers among the top 32 ranks at the French national fencing championships ranged from 35.4% in women’s épée to 33.7% in women’s foil and from 30.2% in men’s épée to 39.6% in men’s foil. Even higher left-hander frequencies were documented among the top-8 (up to 37.5% in women’s épée and 47.2% in men’s foil) and the top-4 (up to 50% in women’s épée and 52.8% in men’s foil). However, while these top echelons showed a higher prevalence of left-handers compared to the overall pool of épée or foil fencers, representing a broader and average performance level, the study was limited by small sample sizes, particularly in the top-4 and to a lesser extent in the top-8 groups. These small samples, coupled with the ranking groups’ lack of independence, compromised the ability to statistically verify significant differences in left-hander proportions between the top-32, top-8 and top-4 groups. In boxing, Raymond *et al*. [10] noted a higher prevalence of left-handers among Danish champions (23.1%) compared to Danish amateurs (6.3%), although this study was also limited by small sample sizes, including only 26 amateur boxers. In tennis, Holtzen [40] found that left-handed players were overrepresented compared to the overall population in higher tiers of the 1999 world rankings in men’s tennis but not in lower ones, assessed in ranking intervals of 50. Extending this research, Loffing *et al*. [41] analysed the prevalence of left-handers in the top-500 male tennis players over nearly four decades (1973−2011). They observed a similar trend of left-handers being more common in the top tiers, particularly at the beginning of their study period. These observations might suggest a distribution pattern similar to figure 1B, indicating a concentration of left-handers in higher performance levels. However, the reliability of some previous findings might be compromised by issues such as the comparison between ranking groups of unequal size, small samples or data limited to local populations and single seasons. To substantiate earlier findings that suggest higher frequencies of left-handers with increasing performance levels, further distributional analyses across a broader range of sports are necessary.

This study aims to build on prior analyses by examining the distribution of left-handed athletes across different performance levels and exploring the relationship between handedness and athletic performance in various sports, including fencing (épée, foil, sabre) and interactive ball games such as table tennis, tennis and badminton. A decrease in left-hander prevalence from higher to lower performance levels, combined with an overrepresentation of left-handers relative to general population estimates—at least in the upper echelons—would support the notion of a performance advantage associated with left-handedness (figure 1B). Conversely, an increase in left-hander prevalence from higher to lower performance levels would challenge the existence of a straightforward link between left-handedness and athletic success (figure 1C). Finally, a lack of correlation could either support (figure 1A) or challenge (figure 1D–F) the notion of an unambiguous performance advantage

## 2. Material and methods

### 2.1. Data retrieval

Sport-specific handedness data for elite athletes were collected across six disciplines in fencing (épée, foil, sabre) and interactive ball sports (table tennis, tennis, badminton). For each discipline, lists of the top-300 senior female and male athletes from year-end world rankings between 2004 and 2023 (badminton: between 2009 and 2023) were retrieved from publicly accessible online databases (table 1). Where applicable, data from previous publications (i.e. [5,8,41]) were incorporated and supplemented with additional data. Information on athletes’ task-specific handedness (i.e. hand used for wielding a weapon or racket) was taken from previous studies, assessed from online databases or determined based on additional searches on the web, such as pictures or videos showing an athlete in action. The complete raw data are made available in the electronic supplementary material.

**Table 1.** Sport-specific online databases for data retrieval.

sport	database
fencing	
épée	www.fie.org
foil	www.fie.org
sabre	www.fie.org
table tennis	www.ittf.com
tennis	www. atptour.com/en, www.wtatennis.com
badminton	https://bwfbadminton.com/

### 2.2. Statistical analysis

For each sport and gender, athletes were ranked based on their highest position in the year-end rankings between 2004 and 2023. Based on these lists, the average proportion of left-handers was calculated across ranks for each sport and gender and compared to the prevalence of left-handers in the general population (females: 9.5%; males: 11.6% [3]) using *χ*² goodness-of-fit tests. To account for multiple comparisons, Benjamini–Hochberg corrections were applied, and the results are presented post-correction.

To investigate the relationship between handedness and performance, Spearman’s rank correlation tests were conducted for each sport and gender to identify potential monotonic trends in left-hander proportions from upper (reflecting higher performance levels) to lower ranks (reflecting lower performance levels). For this analysis, athletes were categorized into ranking groups using ranking intervals of 10, based on their highest position in the year-end world rankings during the respective period: ranks 1−10, ranks 11−20, ranks 21−30 and so on, up to ranks 291−300.

Variation in left-hander frequencies was also assessed and compared between high, medium and low ranking groups using ranking intervals of 100 (ranks 1−100 versus 101−200 versus 201−300) and 50 (ranks 1−50 versus 126−175 versus 251−300). For each ranking group, the prevalence of left-handed athletes was compared to general population estimates of left-handedness using *χ*² goodness-of-fit tests. Additionally, *χ*² tests of independence were performed to examine whether left-hander frequencies varied between high, medium and low ranking groups of the 100-ranking and/or 50-ranking intervals. For both the correlation and the *χ*² tests, Benjamini–Hochberg corrections were used to control for multiple testing (*p*-values are reported post-correction).

For all inferential statistics, the alpha level was set at 0.05. The effect size phi ($\varphi =\sqrt{\chi 2/N}$) was calculated for *χ*^2^ goodness-of-fit analyses, and Cramer’s *V* effect size ($V=\varphi /\sqrt{DF_{\text{min}}}$) was used for *χ^2^* test of independence. Statistical analyses were performed using RStudio 4.4.0.

## 3. Results

Sport-specific handedness was determined for 15 099 senior elite athletes from fencing (épée, foil, sabre) and interactive ball sports (table tennis, tennis, badminton) competing between 2004 and 2023. Among female athletes, all fencing disciplines and table tennis show an average proportion of left-handers across ranks that exceeds the general population estimate (table 2). By contrast, in women’s tennis, the frequency of left-handers across ranks aligns with general population estimates, while it tended to be lower compared to the overall population in women’s badminton (table 2). Among male athletes, the average proportion of left-handers across ranks was higher compared to the overall population in épée, foil and table tennis, but not in sabre fencing, tennis or badminton (table 2).

**Table 2.** Prevalence of left-handed athletes. Frequencies of left-handed female and male athletes listed in the top-300 of year-end world rankings between 2004 and 2023 for fencing (épée, foil, sabre) and interactive ball sports (table tennis, tennis, badminton). Athletes appearing in multiple year-end world rankings were counted only once, based on their highest position in the year-end world rankings during the respective period. Results from *χ*^2^ goodness-of-fit tests compare left-hander frequencies among athletes with those of left-handers in the general population (females: 9.5%; males: 11.6% [3]).

gender	sport	handedness							
		*N*	left	right	NA	% left	*χ* ^2^	*p*	*φ*
female	épée	1667	243	1227	197	16.53	84.515	<0.001	0.240
	foil	2055	411	1487	157	21.65	326.129	<0.001	0.415
	sabre	1237	138	975	124	12.40	10.879	0.001	0.099
	table tennis	1230	170	918	142	15.63	47.475	<0.001	0.209
	tennis	1254	119	1086	49	9.88	0.198	0.657	0.013
	badminton	1211	81	955	175	7.71	3.845	0.060	0.061
male	épée	1816	349	1310	157	21.04	144.073	<0.001	0.295
	foil	1575	384	1039	152	26.99	328.476	<0.001	0.480
	sabre	1334	147	996	191	12.86	1.772	0.275	0.039
	table tennis	1201	268	861	72	23.74	162.205	<0.001	0.379
	tennis	1146	142	1001	1	12.42	1.065	0.362	0.030
	badminton	1080	124	893	63	12.19	0.348	0.555	0.019

### 3.1. Higher left-hander frequencies in higher echelons

**Figure 2.**
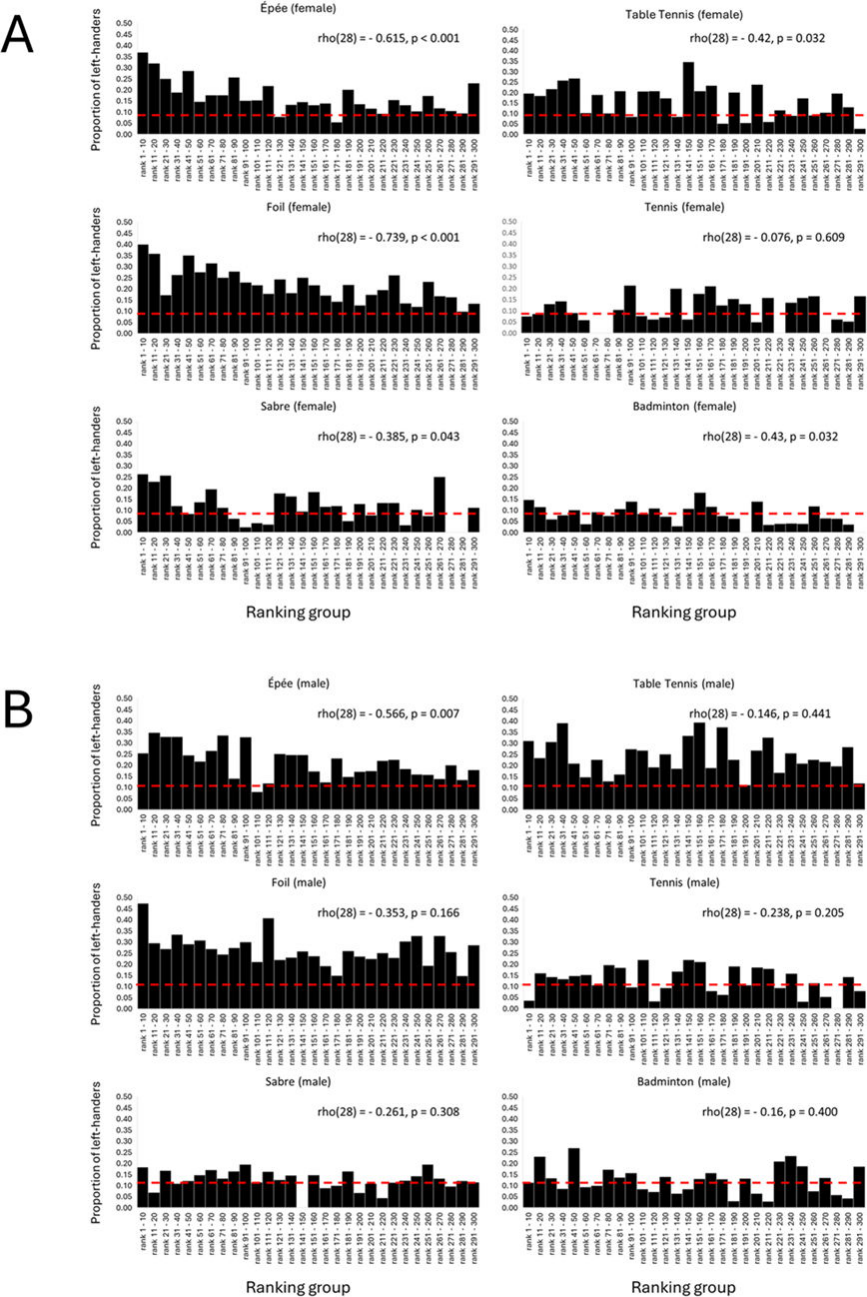
Distribution of left-handed female (A) and male (B) athletes across ranking groups when assessed in ranking intervals of 10. The overall proportion of left-handers in the general population is indicated by the red dashed line (females: 9.5%; males: 11.6% [3]). Spearman’s correlation coefficients with *p*-values (after Benjamini–Hochberg corrections) are given.

In women’s sports, left-hander frequencies were negatively correlated with ranking groups, showing higher proportions of left-handers in upper compared to lower ranks across all disciplines except tennis (figure 2A). Among male athletes, the left-hander proportion was negatively correlated with ranking groups in épée fencing; no correlation was found in the other disciplines (figure 2B).

**Figure 3.**
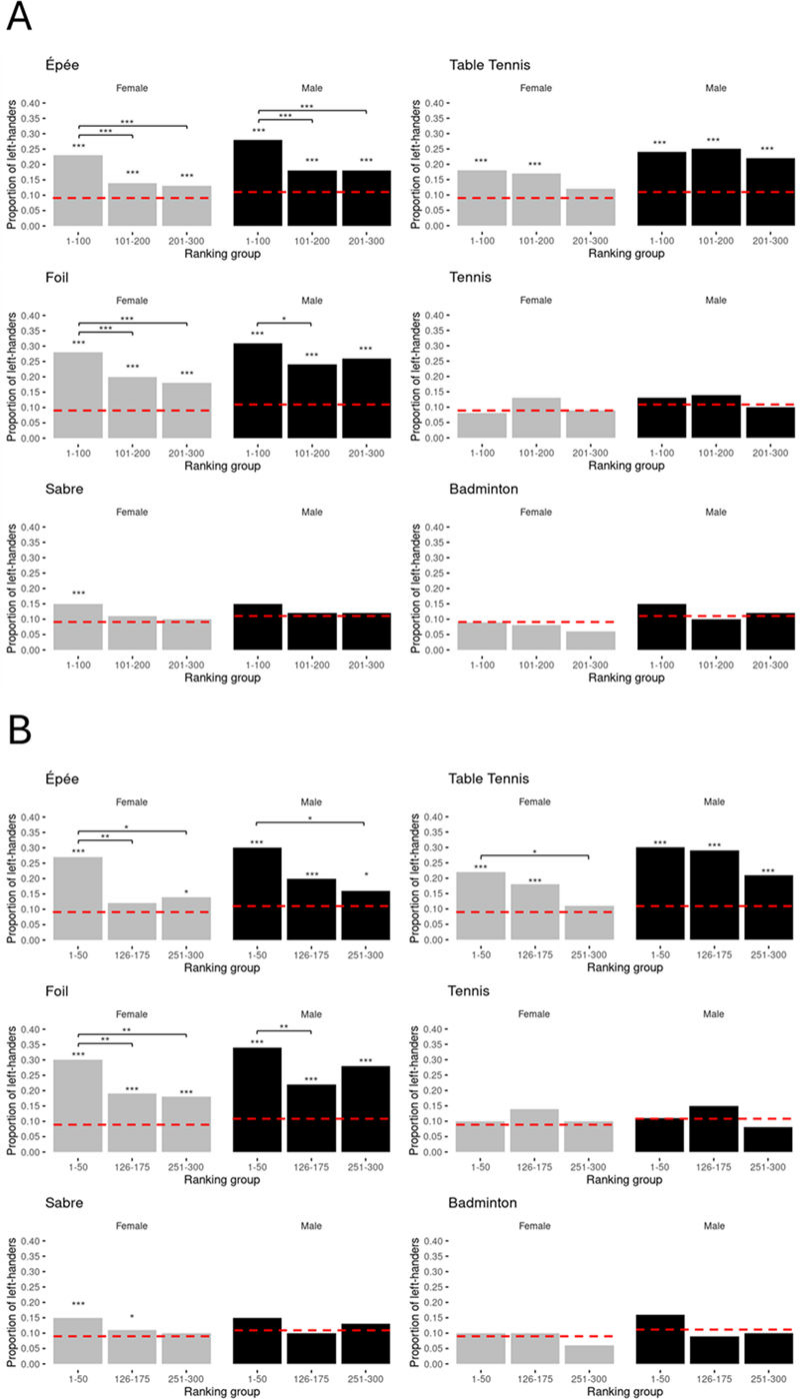
Prevalence of left-handed athletes across high, medium and low ranking groups using ranking intervals of 100 (ranks 1−100 versus 101−200 versus 201−300) (A) and 50 (ranks 1−50 versus 126−175 versus 251−300) (B). The overall proportion of left-handers in the general population is indicated by the red dashed line (females: 9.5%; males: 11.6% [3]). Asterisks indicate statistically significant deviations from overall population estimates and differences in left-hander proportions between different ranking groups within the same ranking interval, respectively (*p < 0.05, **p < 0.01, ***p < 0.001).

Among female athletes, left-handed épée and foil fencers were overrepresented compared to the general population in all ranking groups—high, medium and low—when assessed in intervals of 100 ranks (figure 3A). Female left-handed sabre fencers were only overrepresented in ranks 1−100, but not in ranks 101−200 and 201−300 (figure 3A). In women’s épée and foil, there were relatively more left-handers in the high compared to the medium and low ranking intervals of 100 (all *p* ≤ 0.001, all Cramer’s *V* ≥ 0.100); no differences were found between the medium and low ranking groups (all *p* ≥ 0.418, all Cramer’s *V* ≤ 0.026; figure 3A). In sabre, no differences were found between the different 100-ranking intervals (all *p* ≥ 0.155, all Cramer’s *V* ≤ 0.068; figure 3A). In table tennis, left-handed female players were overrepresented relative to the general population in ranks 1−100 (17.78%, *n* = 360, *χ*² = 28.692, *df* = 1, *p* < 0.001, *φ* = 0.282) and 101−200, but not in 201−300 (electronic supplementary material, table S1). However, no significant differences in left-hander proportions were observed between these ranking groups (all *p* ≥ 0.099, all Cramer’s *V* ≤ 0.078; figure 3A). In women’s tennis and badminton, none of the 100-ranking intervals showed an overrepresentation of left-dominant athletes (electronic supplementary material, table S1), and no differences between these ranking groups were identified (all *p* ≥ 0.097, all Cramer’s *V* ≤ 0.082; figure 3A). Across sports, similar patterns are shown for the 50-ranking intervals (figure 3B). Yet, compared to the 100-ranking intervals, left-handers were not overrepresented relative to the general population in the medium 50-ranking interval (ranks 126−175) in women’s épée fencing and an overrepresentation was found in the medium 50-ranking group in sabre fencing. Moreover, significantly more left-handed female table tennis players were found in the high compared to the low 50-ranking group (*n* = 385, *χ*² = 8.584, *df* = 1, *p* = 0.01, Cramer’s *V* = 0.15). Detailed information on the exact frequencies of left-handed athletes, along with further inferential statistics, can be found in the electronic supplementary material.

Among male athletes, left-handed épée and foil fencers were overrepresented compared to the overall population in all ranking groups—high, medium and low—when assessed in intervals of 100 ranks (figure 3A). In sabre, however, left-hander prevalence did not differ significantly from the general population in any of the 100-ranking intervals (figure 3A). In épée, there were relatively more left-handers in ranks 1−100 than in ranks 101−200 (*n* = 1114, *χ*² = 15.831, *df* = 1, *p* < 0.001, Cramer’s *V* = 0.12) and 201−300 (*n* = 1105, *χ*² = 15.935, *df* = 1, *p* < 0.001, Cramer’s *V* = 0.12; figure 3A). In foil, more left-dominant fencers were found in ranks 1−100 than in 201−300 (*n* = 868, *χ*² = 6.055, *df* = 1, *p* = 0.042, Cramer’s *V* = 0.078; figure 3A). Comparisons between other 100-ranking intervals in épée and foil did not yield significant differences (all *p* ≥ 0.186, all Cramer’s *V* ≤ 0.052; figure 3A). In men’s sabre, no differences were observed between the high, medium and low 100-ranking groups (all *p* ≥ 0.486, all Cramer’s *V* ≤ 0.043; figure 3A). In interactive ball sports, left-handers were overrepresented across all 100-ranking intervals in men’s table tennis (figure 3A); no differences were found between high, medium and low 100-ranking intervals (all *p* = 0.677, all Cramer’s *V* ≤ 0.037; figure 3A). In men’s tennis or badminton, no 100-ranking intervals showed an overrepresentation of left-handers (figure 3A), and no differences were found between ranking groups in these sports (all *p* ≥ 0.207, all Cramer’s *V* ≤ 0.068; figure 3A). Across sports, very similar patterns are seen for the high, medium and low 50-ranking intervals (figure 3B). However, compared to the 100-ranking intervals, the proportion of left-handedness among male table tennis players tended to be higher in the upper two 50-ranking groups compared to ranks 251−300 (all *p* = 0.071, all Cramer’s *V* ≤ 0.068 = 0.1; figure 3B). Detailed information on the exact frequencies of left-handed athletes, along with further inferential statistics, can be found in the electronic supplementary material.

To summarize, the correlation analysis and the investigation of high, medium and low 100- and 50-ranking groups reveal relatively higher proportions of left-handed female athletes in upper compared to lower ranks across all disciplines except tennis. Among male athletes, this pattern is evident only in certain contexts. While higher left-hander proportions are observed at higher echelons in men’s épée fencing and a trend for this pattern is seen in table tennis, the association between handedness and performance-related ranking groups is less clear in foil fencing and absent in sabre fencing, as well as in men’s tennis and badminton.

## 4. Discussion

In this study, we analysed the frequency of left-handed athletes over a 20-year period (2004−2023) across different antagonistic sports disciplines, including fencing (épée, foil, sabre) and interactive ball games (table tennis, tennis, badminton). To further explore the relationship between handedness and athletic performance in these sports, we examined the distribution of left-handed athletes across different ranking intervals representing varying performance levels.

### 4.1. Average (over)representation of left-handers across ranks

Consistent with prior research [5,10,42], our findings confirm a general left-hander overrepresentation across performance levels in épée and foil fencing. By contrast, our results for sabre show a lower overall proportion of left-handers, with an average overrepresentation across ranks noted only among female left-handed fencers—a gender-related difference also reported by Loffing & Schorer [5]. For interactive ball games, our results support earlier work showing an overrepresentation of left-handers in table tennis, but not in tennis or badminton [5,8]. The overlap with earlier work may be partly attributed to our use and extension of the handedness and ranking data reported in those studies. However, our more in-depth distributional analyses further examine the association between left-handedness and relative success in sports, extending beyond previous work.

### 4.2. Increased prevalence of left-handers in higher echelons of fencing and table tennis rankings

To further explore the relationship between handedness and performance among elite athletes, we analysed whether the proportion of left-handed athletes varies between upper (reflecting higher performance levels) and lower ranks (reflecting lower performance levels). We found that most athlete populations with an average overrepresentation of left-handers across ranks also exhibit higher left-hander proportions in upper echelons compared to lower ones. This pattern, with relatively larger proportions of left-handers at upper-ranking group levels, mirrors the skewed distribution illustrated in figure 1B and constitutes strong evidence for a performance advantage of left-handers going beyond what can be inferred from traditional rank-independent analyses of (over)representation.

Our study is not the first to note a higher proportion of left-handers at upper performance levels in certain sports compared to lower levels. However, prior research is sparse and suffers from limitations, such as comparisons between ranking groups that are non-independent and vary significantly in size. For instance, Azémar [6] observed a marked overrepresentation of left-handers across ranks in elite fencing (épée and foil) at French national championships between 1979 and 1998, with a greater number of left-handers in the top-32, top-8 and top-4 (up to 52.8%) than in the total pool of competitors (up to 32.8%). Other reports, like that of Raymond *et al*. [10], are constrained by small sample sizes, comparing left-handedness among only 26 amateur and 95 champion Danish boxers. Compared to previous research, our study used large samples and compared left-handedness between various equally sized, independent ranking groups. Our findings on fencing and table tennis, particularly among female athletes, confirm and expand upon the preliminary findings of earlier work, providing more substantial evidence of a connection between left-handedness and enhanced performance in certain antagonistic sports.

In men’s sabre, as well as in both women’s and men’s badminton and tennis, our data show no evidence that left-handedness confers any advantage in athletic performance. Across these disciplines, the average proportion of left-handers across ranks, as well as the frequencies in high, medium and low ranking groups (analysed in ranking intervals of both 100 and 50), mirrors that of the general population. Notably, women’s badminton suggests a correlation between the frequency of left-handers and athletes’ ranks, with a higher proportion of left-handers at the top echelons. However, since their representation does not surpass that of the general population, there is no clear indication of a competitive benefit for left-handed players. Compared to épée and foil, sabre had generally lower frequencies of left-handers and less evidence of a competitive advantage for them, with some support found in women’s sabre but not in men’s. Lacking evidence for a competitive advantage of left-handed fencers in men’s sabre is consistent with previously published data in the study by Azémar [6] analysing left-handedness among fencers competing in French championships (1979−1998): in men’s sabre, they found an average left-hander proportion across ranks that mirrors general population estimates, with no increased prevalence among the top-32, top-8 or top-4 of fencers either. Differences in left-hander prevalence between épée, foil and sabre may stem from discipline-specific features. Each fencing discipline imposes unique demands on motor control, involves distinct fighting characteristics, utilizes different weapons and has varying target areas; these variations could explain why handedness plays a differing role in achieving high-level success across the fencing disciplines [10,11,14,29]. For tennis, our findings correspond only partially with previous work. While our results do not provide evidence of a link between handedness and performance in tennis, earlier work observed increased left-hander frequencies in higher echelons compared to lower ones. For instance, Holtzen [40] observed an overrepresentation of left-handed male tennis players compared to the general population in ranks 1−50 in the 1999 world ranking, but not in the lower 50-ranking intervals. While these data consider only a single season, a more recent investigation analysed data from men’s tennis over nearly 40 years (1973−2011). More in line with our findings, this longitudinal study found a trend of left-handers being more common in the top tiers only at the beginning of the study period but not in later years [41].

Overall, our findings on varying left-hander frequencies between higher and lower ranking groups indicate a competitive edge of left-handed athletes in fencing and table tennis, but not in tennis and badminton. This may be explained by different discipline-specific spatiotemporal constraints. Specifically, it has been argued that the increased presence of left-handers in fast-paced sports like fencing and table tennis, unlike in relatively slower-paced tennis and badminton, may suggest a distinct advantage for left-handers under conditions of high spatiotemporal pressure [8].

### 4.3. Theoretical implications for the negative frequency-dependent advantage hypothesis versus the innate superiority hypothesis?

Our data from fencing and table tennis, showing higher proportions of left-handed athletes at higher performance levels compared to lower ones, can be explained by the *IS. IS* proposes that left-handedness is linked to certain predispositions that could enhance athletic performance, independent of the rarity of left-handers in the general population. Specifically, it has been suggested that functional specializations of the right hemisphere support more effective left-hand than right-hand movements in (sport-specific) unimanual tasks [11]. Such tasks, which involve dominant right-hemisphere engagement, may include visuospatial, spatiotemporal and visuomotor processing [11,14–18], precision-based aiming activities, such as fencing and racket sports [14,25,26], and object manipulation in close-distance contests, as seen in various combat sports [27,28]. Compared to right-handers, left-handers may also show greater recruitment of both contralateral (opposite to the dominant hand) and ipsilateral (same side as the dominant hand) hemispheric networks during dominant hand use—networks specialized for distinct and complementary motor control processes—potentially conferring an advantage in complex, sport-specific unimanual tasks [29]. Finally, left-handedness has been associated with certain hormone profiles that could contribute to their athletic success [34–37,39]. If left-handedness is associated with such frequency-independent, sport-relevant predispositions, then the extraordinary abilities required at the highest level of competition may indeed make left-handed athletes more common in higher echelons.

Higher concentrations of left-handers at upper-ranking groups can also be explained by the *NFDA*, which maintains that left-handers benefit from their opponents’ reduced familiarity with left-handed action patterns. While the *NFDA* could, in principle, account for our distributional findings, this requires assumptions that may stretch the model’s plausibility. Specifically, if an overrepresentation of left-handers reflects a performance advantage, a stronger overrepresentation at higher performance levels implies an even greater competitive edge over right-handers—compared to lower levels, where this overrepresentation is less pronounced. Under *NFDA* logic, this would imply that athletes at higher levels are either (i) less accustomed to facing left-handed opponents than athletes at lower levels and/or (ii) that unfamiliarity with left-handed opponents has a disproportionately greater impact at the elite level.

Scenario (i) appears implausible. Major fencing and table tennis competitions typically follow a knockout format, where a single loss ends an athlete’s progression (for competitions, see www.fie.org or www.ittf.com). Since left-handers are more prevalent in the upper echelons, the likelihood of encountering left-handed opponents should increase in the later stages of tournaments, where only top athletes remain. Therefore, it is unlikely that high-performing athletes are less accustomed to competing against left-handers. Nevertheless, our findings might still be consistent with *NFDA* if frequency-dependent effects have a greater impact at higher performance levels, as proposed in scenario (ii). At advanced skill levels, athletes’ actions become more variable, and manoeuvres may be executed with greater speed and precision [43,44]. Thus, as skill levels rise, the challenge of anticipating less familiar left-handed movements may grow, potentially reinforcing the left-handers’ advantage. On the other hand, if higher concentrations of left-handers at the top levels also increase the likelihood of facing left-handed opponents in the later stages of competitions, then greater exposure to left-handers may reduce relatively greater frequency-dependent advantages at upper echelons. Moreover, athletes competing at the highest level undergo the most sophisticated and extensive training designed to maximize success [44]. Should left-handers have greater advantages due to negative frequency-dependent effects at the top level, elite-level preparation would likely include specific strategies for competing against them, thereby minimizing disadvantage due to unfamiliarity through targeted adaptation [45–47]. In fencing, for instance, the challenge of facing left-handed opponents has been recognized for centuries; both historical and modern training manuals offer targeted drills to prepare for such encounters [48]. Today, most elite fencing teams include both right- and left-handed fencers, creating ideal internal sparring environments. At the Paris 2024 Olympics, for instance, 10 of the 12 medal-winning national teams in épée and foil included at least one—and up to three—left-handed athletes among their three- or four-person squads (for medal-winning teams, see https://www.olympics.com/en/olympic-games/paris-2024/results/fencing; for athletes’ handedness, see www.fie.org/athletes). Such intra-team handedness diversity could offer regular exposure to left-handed world-class opponents and help reduce unfamiliarity-based disadvantages. A combination of more frequent encounters of high-performance athletes with left-handed opponents and sophisticated professional training seems to challenge the assumption that frequency-dependent effects grow stronger at higher performance levels. Undoubtedly, more research is needed to critically assess *NFDA*’s explanatory power, including more detailed estimates of athletes’ exposure and strategies for adaptation to left-handed opponents. This seems particularly important, as *NFDA* is widely accepted and often favoured over *IS* for explaining left-handers’ edge in certain sports (see §4.4).

### 4.4. Implications for non-antagonistic sports

Previous research has often favoured *NFDA* over *IS* to explain the competitive edge associated with left-handedness in sports [1,10,11]. This preference has been bolstered by data from *non*-antagonistic sports, which do not involve any duel-like interactions, such as javelin throw, shot put, darts or ten-pin bowling. Unlike some antagonistic sports showing a high incidence of left-handed athletes, non-antagonistic sports have been associated with left-hander proportions that align with those of the general population [10,49]. This has been interpreted as evidence against a frequency-independent and general left-handed superiority in sports, thereby challenging *IS*. However, our study underscores the importance of distributional analysis in comparing left-hander proportions between higher and lower performance levels across athlete populations within antagonistic sports. Without a detailed analysis of how left-handedness varies across performance levels, previous conclusions about the irrelevance of handedness for athletic success in non-antagonistic sports may be premature. Therefore, we advocate extending this more in-depth analysis to non-antagonistic sports. Should distributional analyses in non-antagonistic disciplines reveal a concentration of left-handed athletes in the higher performance tiers that exceeds general population levels, this would suggest a performance-relevant role for handedness in these sports as well.

### 4.5. Wider implications for our understanding of human handedness

Apart from shedding light on the role of left-handedness in sports, this study may also contribute to a broader understanding of human handedness. It has been hypothesized that the competitive advantage of left-handers in antagonistic interactions—such as those observed in various sports—was a key factor in the evolution of handedness polymorphism. In (pre)historic populations, where inter-group violence and hand-to-hand combat were common, such an advantage could have translated into increased survival and reproductive success [10,50].

Building on previous research, our distributional findings provide substantial evidence for a performance benefit of left-handers in duel-based sports. This supports the idea that their competitive edge in antagonistic interactions may have conferred fitness advantages in early human contexts. Given that handedness is partly heritable [51], such benefits may have outweighed potential fitness costs in other domains [10]. This trade-off could have led to a stable evolutionary equilibrium, with left-handedness persisting as a minority trait alongside predominant right-handedness.

If left-handedness conferred not only a sporting advantage but also a decisive edge in (pre)historic combat, evolutionary selection may have particularly favoured left-handedness in males, given that males were more frequently involved in antagonistic interactions and warfare [10,50,52]. This could help explain why the prevalence of left-handedness in the overall population is slightly higher in males than in females [3].

### 4.6. Limitations and future directions

Many studies, including the current investigation, have derived information about the (over)representation of left-handers from cross-sectional data aggregating data over a period of several years (e.g. [5,8,10,53,54]). While this approach offers broad insights, it fails to account for potential temporal variations in left-hander prevalence. However, a few studies have incorporated time as a factor, analysing longitudinal sports data that span several decades. For instance, longitudinal data have been published for tennis (1973−2011: [41]), boxing (1924−2012: [55]) and baseball (1957−2005: [56]; 1876−1985: [57]). In men’s tennis, for example, higher frequencies of left-handers have been noted in the top echelons, assessed in 50-ranking intervals, but this trend was only evident at the beginning of the period between 1973 and 2011, not consistently throughout [41]. Among Major League Baseball players, the proportion of left-handed pitchers and batters increased across decades, both stabilizing around 30% from the 1940s onwards. However, left-hander prevalence increased more rapidly among batters than pitchers. More long-term studies on left-handedness and athletic performance are required to explore the factor time. Future research should also explore whether potential fluctuations in left-hander frequencies are the result of changing sports dynamics, training methodologies or recruitment strategies over time. The incorporation of longitudinal analyses into future research is crucial for a more detailed and nuanced understanding of left-handed athletes’ impact on sports across different eras.

This study adopted an international perspective on left-handedness in antagonistic sports. A potential limitation of this approach is that athletes who reach the highest international level have typically passed through multiple selection stages at the national level [58,59]. Handedness may influence these earlier stages of selection, along with cultural factors such as a sport’s popularity, culturally different perspectives on handedness, level of competitiveness and the resulting selection pressure within a country (e.g. [60]). These aspects, however, are often overlooked when focusing solely on international samples, as is the case here. To more thoroughly understand how handedness distributions emerge in antagonistic sports—and to assess how well they align with *NFDA* and/or *IS*—future research should incorporate national and cross-cultural comparative perspectives.

Previous studies on left-handers’ roles in one-on-one interactive sports have not considered athletes’ experience levels in competing against left-handers. To better understand the mechanisms behind left-handers’ advantages in antagonistic contexts, future research should include data on athletes’ experience with left-handed opponents. We hypothesized that high-performance athletes likely receive sophisticated training, which includes specific exposure to left-handed competitors [45–47]. If it is found that a higher concentration of left-handers at elite levels coincides with greater familiarity among top athletes with competing against them, this could challenge *NFDA*.

Our results highlight gender disparities in sports, with men’s sports typically featuring higher proportions of left-handers, while women’s sports show more pronounced differences in left-hander frequencies between higher and lower performance levels. These disparities could be due to differences in tactics and dynamics characteristic of specific sport disciplines. For instance, gender-related variations in physical constitution and athleticism might influence the pace of sport-specific interactions, such as the speed of rallies in interactive ball games. This, in turn, could create different spatiotemporal pressures and demands for perceptual anticipation of an opponent’s actions, as well as the sensory-motor integration required to counter an opponent’s moves. Such differences may confer competitive advantages on left-handed athletes in gender-specific ways. Additionally, the link between enhanced sport-relevant psychomotor skills and handedness could also differ by gender, potentially contributing to these disparities. However, the factors underlying gender differences in the representation of left-handed athletes in competitive sports remain unclear and warrant further investigation.

## 5. Conclusion

In fencing (épée, foil, sabre) and table tennis, we found an average overrepresentation of left-handers across ranks and higher left-hander proportions in upper echelons compared to lower ones. While this pattern was particularly seen in women’s sports, it was less pronounced but still evident in men’s sports.

Higher concentrations of left-handed athletes in higher echelons provide clear evidence for a link between left-handedness and athletic success, going beyond what traditional rank-independent analyses of (over)representation alone suggest. While our distributional data are compatible with the widely accepted *NFDA*, we have also raised the question of whether left-handers’ performance edge might additionally involve frequency-independent factors, as suggested by the often-overlooked *IS*. Importantly, our findings do not allow us to favour one explanation over the other. The underlying causes are potentially multifactorial, involving both rarity-related and possibly frequency-independent factors, and require further targeted research to be disentangled.

## Data Availability

The raw data are provided as supplementary material [61].
